# Graphene Oxide Nanoparticle-Assisted Promotion of Stevioside, Rebaudioside A, and Selected Biochemical Attributes in *Stevia rebaudiana* Bertoni

**DOI:** 10.1155/2024/6693085

**Published:** 2024-05-31

**Authors:** Muhammad Talha Rafiq, Zahoor Ahmad Sajid, Sheza Ayaz Khilji

**Affiliations:** ^1^Plant Developmental and Regenerative Biology Laboratory, Institute of Botany, University of the Punjab, Quaid-e-Azam Campus 54590, Lahore, Pakistan; ^2^Department of Botany, Division of Science and Technology, University of Education Township, Lahore, Pakistan

## Abstract

*Stevia rebaudiana* Bert. is commonly known as candy leaf, sugar leaf, or sweet leaf. It is a natural sweetener that has low calories and is used as a substitute for sucrose. The objective of this research is to evaluate the effects of graphene oxide (GO) on the growth, biochemical activities, and stevioside and rebaudioside A production of *Stevia* in in *vitro-*raised plantlets. For this, green nanomaterials of GO (0, 2, 4, 6, 8, and 10 mgL^−1^) were applied to the *in vitro* plants to enhance its sweetness by triggering the production of stevioside and rebaudioside A and other growth and biochemical parameters. It was observed that all the growth parameters of *Stevia* plants significantly increased with all GO treatments tested. Total chlorophyll and protein contents were increased (1.85- and 2.65-fold increase from the control) by applying 8 mgL^−1^ of GO to the MS medium. The maximum value (4 mg·g^−1^ of protein) of peroxidase activity (POD) was observed by applying 4 mgL^−1^ of GO, 28.92-fold increase from the control. In comparison, superoxide dismutase activity (SOD) (0.4 mg·g^−1^ protein) was observed with 10 mgL^−1^ of GO (1.56-fold increase from the control). Stevioside (12.9 and 8.9 mg·g^−1^ DW) and rebaudioside A (3.2 and 0.81 mg·g^−1^ DW) were observed only at 6 and 8 mg·L^−1^ treatment of graphene oxide. According to the findings, using graphene oxide (GO) had a significant impact on the growth, biochemical activities, and steviol glycoside production in *Stevia*. This shows that GO has the potential to be a valuable enhancer of sweetness and overall *Stevia* leaf quality, providing great prospects for the development of low-calorie natural sweeteners.

## 1. Introduction

*Stevia rebaudiana* Bert. is a perennial herb that belongs to the *Asteraceae* family. It is one of the only two known species of the genus *Stevia* that produce steviol glycosides, i.e., rebaudioside A, stevioside, and rebaudioside C, found in *Stevia* leaves. It is native to Paraguay, Brazil, and South America [1–3]. This natural sweetener due to having low calories is used as a substitute for sucrose all over the world [4]. Steviol glycosides (SGs), such as stevioside and rebaudioside A, are major sweetening compounds found in *Stevia* leaves, which are considered natural and noncaloric sweeteners [5]. Steviol glycosides make this plant almost 100–300 times sweeter than sucrose, offering a promising solution to the global rise in obesity, diabetes, and other related health issues [1, 6]. *Stevia* leaves are also enriched with vitamins, minerals, fatty acids, and essential amino acids. Moreover, *Stevia* is also a good source of various bioactive compounds, namely, flavonoids, folic acid, phenolic compounds, chlorogenic acids, hydrocarbons, and crude fiber, all having enormous health benefits [7–9]. *Stevia* plant possesses anti-inflammatory, antioxidant, and antimicrobial properties. These properties contribute to various health advantages, including the prevention and management of chronic diseases such as cardiovascular diseases, obesity, and metabolic syndrome [10–12]. Because *Stevia* leaves have anti-inflammatory and antioxidant qualities, they are used in a variety of products despite their inherent sweetness. Their potential to manage hypertension, diabetes, and obesity is being investigated and authenticated in several studies. This research work can help to establish a quick *in vitro* propagation technique with higher steviol contents, which will be advantageous to the farming sector [13]. The increasing demand for natural noncaloric sweeteners has led to a search for alternative sources to replace traditional sugar. In addition to the promising health benefits, approval of *Stevia*'s use as a sweetener by the Food and Drug Administration (FDA) in 2011 [14] further increased its demand worldwide both in the food and pharmaceutical industries. However, the low yield of steviol glycosides and the high cost of production limit in commercial applications of *Stevia*. Therefore, there is an urgent need to enhance the production of steviol glycosides (SGs) in *Stevia* plants. One promising approach is the use of elicitors under *in vitro* conditions, which lead to the production of secondary metabolites with potential health benefits [15, 16]. The plant tissue culture technique has its unique potential to establish a sustainable system not only to multiply the plant but also to produce medicinally important PSM in limited time, space, and resources [17, 18]. Graphene oxide (GO) is undoubtedly just one of the many elicitors used to improve plant responses and activate the formation of secondary metabolites in different plant systems. For their capacity to evoke certain responses in plants, several different elicitors have been the subject of in-depth research. Salicylic acid (SA) stands out as a notable example among them and is well known for playing a crucial part in the defense mechanisms of plants. Increased resistance to biotic stresses such as infections and pests is promoted by the efficient induction of defense-related genes [19]. Methyl jasmonate (MeJA), another well-known elicitor, functions in plants as a signaling molecule, activating the jasmonic acid pathway and triggering the creation of secondary metabolites such as terpenoids and phenolics [20]. The abovementioned elicitors have been used to support plant responses to environmental stresses and to increase the production of valuable bioactive chemicals, along with others such as chitosan, abscisic acid (ABA), and *meta*-topolin and even microbial-based elicitors [9]. The adaptability and potential of elicitors in contemporary agriculture and biotechnology are shown in the fact that the choice of elicitors frequently depends on the particular plant species, the targeted metabolites, and the environmental circumstances in which the plants are cultivated [21].

Graphene oxide, a two-dimensional carbon nanomaterial, has demonstrated remarkable elicitor properties in various plant systems. In plant cells, GO can stimulate the generation of reactive oxygen species (ROS). In response to stress, ROS serves as a signaling molecule and activates the plant's defense mechanisms [22]. Graphene oxide (GO) has shown potential in enhancing the production of bioactive compounds and promoting plant growth [23, 24]. GO enhances plant growth by promoting root growth and photosynthesis [25]. Nanoparticles (NPs) behaving as abiotic stress stimulators evoke the defense mechanism of plants, eventually resulting in the improved production of secondary metabolites (SMs) [26]. Similarly, GO NPs significantly enhance the production of plant SMs by promoting the activity of enzymes which in turn improves the production of SMs [24]. The unique physicochemical properties of GO, such as its large surface area, high reactivity, and ability to interact with biological molecules [27], make it an intriguing candidate for eliciting the production of steviol glycosides in *Stevia*.

According to our knowledge, although studies have been reported regarding the elicitation of glycoside in *Stevia* but information regarding the use of graphene oxide as an elicitor is scanty and the need is still there to explore the potential of this biomolecule, especially in the form of nanomaterial. So, this study was carried out to observe the effect of GO on the elicitation of steviol glycoside in *Stevia* under *in vitro* conditions. Due to their distinctive characteristics at the nanoscale, nanoparticles are essential in many applications, including elicitation investigations. Our findings offer an insight into the logical design of an effective nanomaterial-assisted culture system that may be utilized to speed up both plant growth and the production of steviol glycosides in *Stevia* plants with the use of graphene oxide.

## 2. Materials and Methods

### 2.1. Plant Materials and Preparation of Media Containing Different Concentrations of Graphene Oxide Nanoparticles

Already raised germplasm of *Stevia rebaudiana* (collection no. Bot-315) in the Plant Developmental and Regenerative Biology Laboratory, Institute of Botany, University of Punjab, Lahore, Pakistan, was used to raise *in vitro* plants that were used as an explant source for this study. Morishige and Skoog (MS) medium [28] without any plant growth regulator was used to propagate this plant under 16/8 hour light/dark (32 *µ*mol·m^−2^s^−1^) period and 25 ± 2°C temperature [29]. These *in vitro* plants were subcultured after every 15 days to get a reasonable number of plants to set up further experimentation. This experiment was carried out in the year 2022-23 in the Plant Developmental and Regenerative Biology Laboratory, Institute of Botany, University of Punjab, Lahore, Pakistan (31° 30′ 15″ North, 74° 18′ 23″ East). MS basal medium containing 0.7 *µ*M IBA with 6 different treatments (Table 1) of graphene oxide was prepared to see their effect on *Stevia rebaudiana* for its growth and to enhance steviol glycosides. Nodal segments (*ca*., 1 cm) were used as an explant during this investigation. For each treatment, 16 replicate culture vessels were inoculated on an MS medium containing different concentrations of GO. The nodal segment inoculated on MS medium plus 0.7 *µ*M IBA without GO was considered as control. All of the culture tubes were tagged and incubated under control conditions of light (32 *µ*mol·m^−2^s^−1^) using 40 W white florescent tube rods at a temperature of 25 ± 2°C. Graphene oxide nanoparticles (green synthesis) prepared by Hummer's technique [30] was generously provided by Zill-e-Huma Aftab, Department of Pathology, Faculty of Agricultural Sciences, University of Punjab, Lahore, Pakistan. The size of GO nanoparticles can range from a few nanometers to several micrometers, and they have a two-dimensional structure. A thin sheet-like structure with a hexagonal lattice is typical of GO nanoparticles. GO nanoparticles have oxygen-containing functional groups, such as carboxyl (-COOH), epoxy (-O), and hydroxyl (-OH) groups, on their surfaces [31]. To maintain stability and uniformity during application, the nanoparticles may be dispersed in a specific solvent or medium [32].

### 2.2. Data Collection for Various Growth and Biochemical Parameters

Plants were allowed to grow at these treatments for 60 days and the data were collected for various growth parameters as well as the chlorophyll content, soluble protein contents, antioxidant activities (peroxidase and superoxide dismutase activities), and SG contents by high performance liquid chromatography (HPLC).

#### 2.2.1. Morphological Observation

Different morphological observations, *viz.,* number of leaves, roots, and nodes; length and width of leaves (cm); length of roots, stems, and plant (cm); fresh weight of stem, leaves, and roots (g); and dry weight of stem and leaves (g) were measured at day 60 of the GO treatment.

#### 2.2.2. Chlorophyll Contents' Analysis

For chlorophyll extraction, 0.05 g leaves were incubated in 5 ml of dimethyl sulfoxide (DMSO) under dark at 25 ± 2°C for 72 h. The absorbance of chlorophyll extract was observed at 663 and 645 nm using a spectrophotometer (UV 4000 Hamburg, Germany). Total pigment contents were calculated by optical density at 663 (D663) and 645 nm (D645) as shown in the following equation [33]:

(1) $$\begin{array}{c} \text{Total Chl }\left(\frac{\text{mg}}{g}\right)=\frac{0.00802\times \text{OD}663+0.0202\times \text{OD}645\times V}{W}, \end{array}$$

where OD is the optical density, *V* is the volume of the sample, and *W* is the weight of the sample.

#### 2.2.3. Protein Content and Antioxidant Activity

One gram of the plant material was cryogenically ground and mixed with 2 ml of 1 M phosphate buffer (pH 7.2) and 0.1 g of *polyvinyl-pyrrolidone* (Sigma-Aldrich). After thorough mixing, the slurry was centrifuged at 14000 rpm at 4°C for 30 minutes. The resulting supernatant was collected and stored at −20°C to use for protein and antioxidant enzyme analysis.

#### 2.2.4. Analysis of Total Soluble Proteins

The estimation of soluble protein contents was done by the Biuret method of Racusen and Johnstone [34]. Biuret reagent (2 ml) was mixed in 0.2 ml of supernatant and for control, 0.2 ml distilled water was taken instead of the supernatant. The optical density for total dissolved proteins was taken at 545 nm with a spectrophotometer. The protein content was calculated from a standard curve of bovine serum albumin as shown in the following equation:

(2) $$\begin{array}{c} \text{Protein content}\left(\frac{\text{mg}}{g}\right)=\frac{\text{CV}\times \text{TE}}{\text{EU}\times \text{Wt}\times 1000}, \end{array}$$

where CV is the curve value, TE stands for total extract, EU is the extract used, and Wt is the fresh weight of the sample.

#### 2.2.5. Peroxidase Activity (POD)

The method of Racusen and Foote [35] was used for POD analysis [20]. For this, 10 *µ*l of enzyme extract and 2.5 ml of 0.1 M phosphate buffer (pH 7.2) were added in both experimental and control tubes for enzyme assay. Finally, 0.2 ml of guaiacol was added in the experiment; however, in control, 0.2 ml of distilled water was added instead of guaiacol. Both experimental and control tubes were kept for 30 minutes followed by the addition of 0.1 ml of 0.3% H_2_O_2_ in both, i.e., control and the experimental one. The optical density was measured at 470 nm using spectrophotometer (equation (3)).

Peroxidase activity (mg/g protein) = A × df/EU × Wt × 1000 (equation (3)), where *A* = absorbance, df = dilution factor, EU = extract used, and Wt = fresh weight of the sample tissue.

#### 2.2.6. Analysis of Superoxide Dismutase (SOD) Activity

The analysis of superoxide dismutase activity was done by using Maral et al. [36] method with certain modifications. Two samples of experimental and control were made. Experimental samples were made with 20 *μ*l of the enzyme extract and 2 ml of the reaction mixture. However, the control samples contained only the reaction mixture. Both the samples were placed under white fluorescent light (30 W) for 30 min and absorbance was estimated at 560 nm.

(3) $$\begin{array}{c} \%inhibition=\frac{\text{Absorbance of control sample}-\text{Absorbance of experimental sample}}{\text{Absorbance of experimental}}\times 100. \end{array}$$

### 2.3. Sample Preparation and Conditions for HPLC Analysis of Steviol Glycoside

#### 2.3.1. Sample Preparation for HPLC Analysis

Dried leaves powder was weighed (0.50 g) and added with 25 mL 60% ethanol in water. The solution was extracted using an ultrasonic homogenizer at 40°C for 15 min. The solution was filtered using filter paper, and the filtrate was collected. The residue was re-extracted using 25 mL 60% ethanol. This step was repeated three times, and the filtrate was collected. The filtrate volume was added to 100 mL in the flask using the same solvent. The final solution was filtered with a 0.45 *µ*m microfilter before being injected into the column.

#### 2.3.2. HPLC Conditions and Use of Standard

The stationary phase used was Eurosphere C-18 (250 × 4.6 mm, 5 *µ*m), while the mobile phase used was the mixture of water-methanol (90 : 10; *v/v*) adjusted to pH 3.0 with phosphoric acid, acetonitrile, and TFA in the ratio 65 : 35 : 0.01 (*v/v*). The mobile phase homogenization was conducted by sonification for 30 minutes. The column temperature was maintained at 30°C. The flow rate of the mobile phase was 0.6 mL/min. The detection was made with a UV detector at a wavelength of 210 nm [37]. The volume injected was 20 *µ*L using Rheodyne 7726i injector. The standard of stevioside and rebaudioside A was purchased from Carbosynth Ltd. and Sigma-Aldrich. Chemicals, respectively for high-performance liquid chromatography. Methanol phosphoric acid, acetonitrile, and trifluoroacetic acid (TFA) were purchased from Sigma-Aldrich. All the chemicals used in this investigation were of scientific grade. The formula to calculate the concentration of stevioside and rebaudioside A using HPLC is shown in the following equation:

(4) $$\begin{array}{c} \%\;\frac{Area\;of\;sample\times concentration\;of\;standard\left(mg\backslash ml\right)}{Average\;area\;of\;standard\times concentration\;of\;sample\;\left(mg\backslash ml\right)}\times 100. \end{array}$$

### 2.4. Statistical Analysis

All *in vitro* experiments were conducted as a completely randomized design. Fifteen replicate culture vessels were inoculated for each treatment and an experiment was repeated three times. The Duncan multiple range test was employed [38] to the data using SPSS version 21.0 to compare the means ± standard error (SE) to see the effect of various treatments of GO nanoparticles on various growth and biochemical parameters.

## 3. Results

The application of different treatments of graphene oxide significantly influenced the morphological and biochemical parameters of *Stevia rebaudiana*.

### 3.1. Effects of Graphene Oxide Treatments on Morphological Parameters of *Stevia*

**Figure 1:**
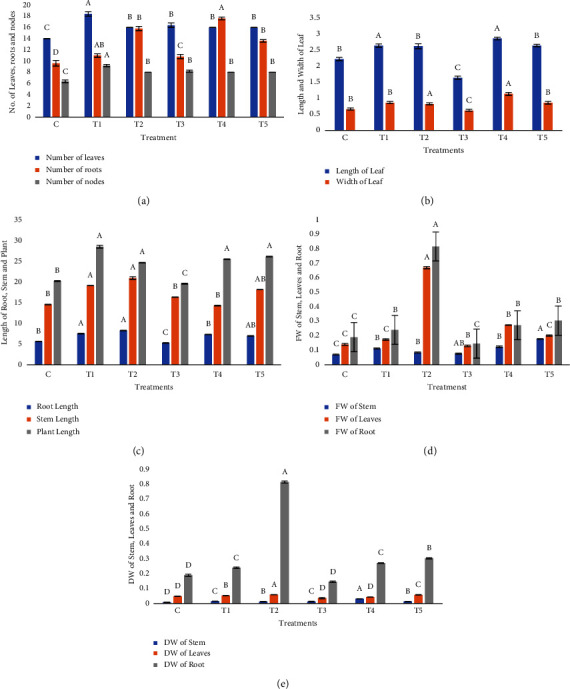
(a) Effects of different concentrations of graphene oxide treatments on the number of leaves, roots, and nodes; (b) length and width of leaves (cm), (c) length of root, stem, and plant (cm); (d) fresh weight of stem, leaves, and roots; (e) dry weight of stem, leaves, and roots in *S. rebaudiana*. Bars with different alphabetical letters showed significant differences and with similar letters showed a nonsignificant effect of different concentrations of graphene oxide treatments on morphological parameters in *S. rebaudiana* according to DMRT at *p* < 0.05.

Different GO concentrations were applied to the *Stevia* plant, resulting in significant effects on morphological parameters in comparison to the controls. The leaf numbers were the highest (18) at T1 while the control treatment had the lowest (14). The number of roots also increased significantly, at T4 (17.6), surpassing controls (9.6). In the case of node number, all the tested GO concentrations showed an increase in a number of nodes, particularly at T1 (9.2), as compared to control plants with 6.4 nodes per plant. Similarly, T4 had the maximum increase in leaf length (2.86 cm) followed by T2 (2.62 cm) as compared to control plants (2.22 cm) and, hence, showed a significant difference in GO-treated vs. nontreated plants. In this study, T2 treatment showed the maximum root and stem length as 8.32 and 28.56 cm, respectively, in comparison to control plants. In case of T3 treatment of GO, root length (5.3 cm), plant height (19.64 cm), and stem length (16.38 cm; Figure 1) were observed.

The addition of GO (all treatments) in MS medium resulted in a significant increase in the fresh weight of the plant, with T5 treatment exhibiting the maximum increase in fresh weight of the stem (0.178 g), while T2 demonstrated the maximum increase in fresh weight of leaves (0.67 g) and roots (0.816 g). The control plants, on the other hand, showed the minimum fresh weight values for all plant parts. So, there was a significant (*P* < 0.05) difference in treated and nontreated plants of *Stevia*. Similarly, dry weight was also increased with all the tried concentrations of GO; the maximum increase in stem dry weight (0.0323 g) was observed in T4 treatment, whereas the maximum increase in dry weight of the leaf (0.0598 g) and root (0.816 g) was recorded with T2 treatment. Control plants, on the other hand, had the minimum dry weight values for all plant parts. The lowest stem dry weight (0.01294 g) was observed at T2, while leaf dry weight (0.0434 g) was recorded at T4 treatment of GO. At T1 treatment, minimum (0.24 g) root dry weight as compared to controls was observed. Overall, results of the present investigation demonstrated the significance difference of GO in increasing the biomass of various plant morphological attributes of *Stevia* plants grown under *in vitro* conditions (Figures 1(a)–1(e)).

### 3.2. Effects of Graphene Oxide Treatments on Biochemical Parameters of *Stevia*

#### 3.2.1. Chlorophyll a and b

**Figure 2:**
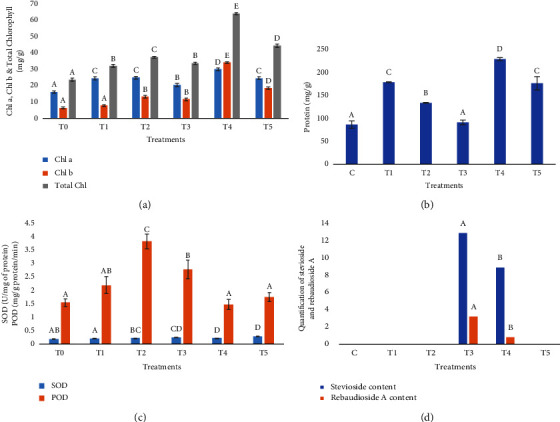
(a) Changes in chlorophyll a, chlorophyll b, and total chlorophyll (mg/g) contents; (b) total soluble protein content (mg g^−1^); (c) SOD (U/mg of protein) and POD (mg g^−1^ protein/min); and (d) stevioside and rebaudioside A content in *S. rebaudiana* at different treatments of GO. Bars with different alphabetical letters showed significant differences and with similar letters showed a nonsignificant effect of different concentrations of graphene oxide treatments on biochemical parameters of *S. rebaudiana* according to DMRT at *p* < 0.05.

The application of GO at various concentrations showed a significant effect on chlorophyll “a” and “b” content in *Stevia* as compared to control plants. The minimum mean value (16.15 mgg^−1^) of chlorophyll “a” was observed in control plants while maximum increase (29.91 mgg^−1^) was observed with T4 followed by T3 treatment of GO (20.47 mgg^−1^). Same as in the case of chlorophyll a, chlorophyll b also showed an increasing trend with GO treatment, and maximum chlorophyll “b” content (33.95 mgg^−1^) was observed by treating plants with T4 as compared to control plants (6.48 mg·g^−1^). Different concentrations of GO were applied to *Stevia* plants, showing that the total chlorophyll content was the highest (63.47 mgg^−1^) at T4 as compared to control plants (23.62 mgg^−1^). The minimum increase in the chlorophyll content was observed at T1 (31.83 mgg^−1^). Hence, the addition of GO in MS medium to *Stevia* plants increased the total chlorophyll content significantly as compared to control plants (Figure 2(a)).

### 3.3. Total Soluble Protein Contents

The lowest value of protein contents was observed in control plants. However, when GO was added to the MS medium, it significantly enhanced the total soluble protein contents. The protein content was increased with its maximum value at T4 (216.92 mg·g^−1^) as compared to control plants (81.81 mg·g^−1^). Protein contents at T3 treatment of GO was 86.26 mg·g^−1^ followed by T2 treatment. Overall, the results showed that the addition of GO to *Stevia* increased the protein content significantly as compared to control plants (Figure 2(b)).

### 3.4. Effects of Graphene Oxide Treatments on Antioxidant Activities of *Stevia rebaudiana*

#### 3.4.1. Superoxide Dismutase (SOD) and Peroxidase (POD) Activities

The results demonstrated that *Stevia* plants grown on the MS medium fortified with different concentrations of graphene oxide (GO) greatly boosted the superoxide dismutase and peroxidase activities as compared to control plants. The highest SOD activity (0.28 U·mg^−1^ of protein; 1.56-fold increase from the control) was observed at T5 followed by T3, whereas T1 showed the lowest increase in SOD activity (0.20 U·mg^−1^ of protein) as compared to the control with 0.18 U·mg^−1^ of protein. Similar, as in case of SOD, the highest peroxidase activity (3.78 mg·g^−1^ protein/min; 28.92-fold increase from the control) was observed at T2 treatment followed by T3 and T1 treatment. T4, on the other hand, had the lowest peroxidase activity (0.13 mg·g^−1^ protein/min). From all the tested GO concentrations, T5 had the minimum increase in the peroxidase activity, with a mean value of 1.72 mg·g^−1^ protein/min. These findings demonstrated the significant impact of GO on antioxidant activities of plants grown under *in vitro* conditions (Figure 2(c)).

### 3.5. Quantification of Stevioside and Rebaudioside A from the Leaves of *S. rebaudiana*

**Figure 3:**
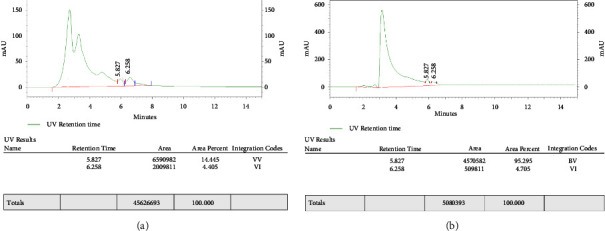
(a) Graphical representation of T3 concentration of graphene oxide treatments on stevioside and rebaudioside A content in *S. rebaudiana*. In graph, 5.827 minutes is the retention time for stevioside and 6.258 minutes is the retention time for rebaudioside A as compared to the standard. (b) Graphical representation of T4 concentration of graphene oxide treatments on stevioside and rebaudioside A content in *S. rebaudiana.* In graph, 5.827 minutes is the retention time for stevioside and 6.258 minutes is the retention time for rebaudioside A as compared to the standard.

**Figure 4:**
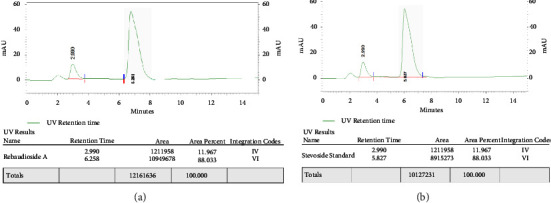
(a) Graphical representation of the standard of rebaudioside A having retention time 6.258 minutes in HPLC chromatograph and (b) graphical representation of the standard of stevioside having retention time 5.827 minutes in HPLC chromatograph.

The amount of SGs in leaves varied greatly, including stevioside and rebaudioside A. Nanoparticles treated cultures with 6.0 mgL^−1^ of GO contained 12.9 and 3.2 mg·g^−1^of DW of stevioside and rebaudioside A, respectively. The other medium treated with 8.0 mgL^−1^ GO showed stevioside (8.9 mg·g^−1^of DW) and rebaudioside A (0.81 mg·g^−1^ of DW) while for other treatments, stevioside and rebaudioside A contents were not observed (Figures 2(d), 3(a), 3(b), 4(a), and 4(b)).

## 4. Discussion

The effects of graphene nanoparticles on higher plants have been extensively studied, and its beneficial impacts on plant development and stress tolerance was recorded when used in low doses [39]. To determine its impact on growth and glycoside production, we treated *in vitro* plants of *Stevia* with GO, and it was found that exposure to 8 mg/L GO in the MS medium increased all growth parameters of *Stevia* plants in comparison to control plants. As compared to the plant inoculated on MS media with GO fortification, the number of leaves and roots reached their maximum value at T1 and T4, respectively. These outcomes are consistent with those of Dimkpa et al. [40] who found that GO nanoparticles have the ability to activate metabolic pathways and produce active constituents for plant metabolism. Similarly, Guo et al. [41] observed that quinoa seedlings with different GO concentrations showed an increase in shoot/root growth. Begum et al. [42] proposed a major role of GO in encouraging tomato seedlings and mature plants to accumulate roots and biomass in a different study. In comparison to control plants (0.19 g), T2-treated plants showed the highest increase in root fresh weight (0.81 g), and the similar trend was observed for root dry weight (0.81 g). These outcomes support the previous research by Zhou et al. [43] who suggested that GO has the ability to improve plant growth, photosynthesis, and nutrient levels significantly. The fact that plant components including leaves, roots, nodes, and fresh and dry weight promotion suggest that GO may improve nutrient intake or increase hormones that promote growth [29]. These changes may be caused by the unique properties of graphene oxide, such as its vast surface area and functional groups, which may enhance growth conditions by promoting water retention or nutrient adsorption [23].

Chlorophyll and protein content were positively impacted by graphene oxide supplementation. Total chlorophyll concentration increased significantly in T4 as compared to control (increased from 23.62 mg·g^−1^ to 63.47 mg·g^−1^). When GO was added in the MS medium, T1 showed the least amount of rise (31.83 mg·g^−1^) in comparison to all other treatments. GO may either improve light absorption or protect chlorophyll molecules from degradation, both of which are critical for photosynthesis, if its chlorophyll content rises, which is consistent with Chen et al. [44] in pea plant. Using graphene oxide nanoparticles, Nokandeh et al. [45] also observed increased levels of proteins, soluble carbohydrates, flavonoids, and phenols. During this study, supplementing the MS medium with graphene oxide also raised SOD activity significantly (0.28 U/mg protein) at T5 treatment when compared to the control plants (0.18 U/mg protein). T1 showed the lowest increase (0.20 U/mg protein) in SOD as compared to the control. The peroxidase activity of T2 was the highest (3.78 mg/g protein/min), as compared to control, suggesting that GO affects the antioxidant activity significantly. Their increased activity might be the consequence of the antioxidant defense mechanism of the plant being triggered in reaction to a mild stress response brought on by GO. The plant may be better able to tolerate stress in general and oxidative damage may be decreased as a result of this increase in the antioxidant enzyme activity [46].

The most notable finding of this study is that GO has distinct impacts on the synthesis of stevioside and rebaudioside A, the two primary sweet ingredients in *Stevia*. Exhibiting the maximum concentration of these compounds, the plants treated with 6.0 mg·L^−1^ of GO indicated the ability of GO to promote secondary metabolite production, which is consistent with the study by Yadav et al. [47]. This might be a result of the stress response because plants are known to produce more secondary metabolites in response to stress [26]. The results of Javed et al. [48] and Dey [49] also confirmed that steviol glycoside synthesis was improved by nanoparticles and that antioxidant activity was increased by using GO. In the work by Rivero-Montejo et al. [16], the impact of graphene nanoparticles as elicitors on *Stevia rebaudiana* is investigated and reported that GO significantly enhanced steviol glycoside.

## 5. Conclusion

The application of 6 and 8 mgL^−1^ graphene oxide (GO) demonstrated a significant positive impact on the growth, biochemical activities, and steviol glycoside production in *Stevia rebaudiana* plantlets. This research highlights the potential of GO as a valuable biomolecule for enhancing the sweetness and overall quality of *Stevia* plants, which could have promising implications for the production of this natural sweetener with a low-calorie content. Significant new lines of inquiry have been opened by this study that demonstrated how graphene oxide (GO) can be used to improve the growth and biochemical characteristics of *Stevia rebaudiana* Bertoni. Further exploration of optimal GO concentrations and cultivation conditions is warranted for the sustainable development of *Stevia* as a sugar substitute. Molecular and cellular mechanisms underpinning GO's impact on growth, photosynthesis, and secondary metabolite production are the focus of these areas, which aim to provide a thorough understanding of GO's interactions with plant systems. In the future, research can take advantage of GO's revolutionary potential in agriculture by focusing on these areas, which will improve crop nutritional quality, sustainability, and productivity.

## Figures and Tables

**Table 1:** Treatments of graphene oxide nanoparticles added in the MS medium containing IBA.

Medium	Concentration of graphene oxide
C	Control (MS basal medium) + 0.7 *µ*M IBA
T1	MS + 0.7 *µ*M IBA + 2 mg·L^−1^ GO
T2	MS + 0.7 *µ*M IBA + 4 mg·L^−1^ GO
T3	MS + 0.7 *µ*M IBA + 6 mg·L^−1^ GO
T4	MS + 0.7 *µ*M IBA + 8 mg·L^−1^ GO
T5	MS + 0.7 *µ*M IBA + 10 mg·L^−1^ GO

## Data Availability

The data used to support the findings of this study are included within the article.
